# Characterisation of the *Candida albicans* Phosphopantetheinyl Transferase Ppt2 as a Potential Antifungal Drug Target

**DOI:** 10.1371/journal.pone.0143770

**Published:** 2015-11-25

**Authors:** Katharine S. Dobb, Sarah J. Kaye, Nicola Beckmann, John L. Thain, Lubomira Stateva, Mike Birch, Jason D. Oliver

**Affiliations:** 1 F2G Ltd., Lankro Way, Eccles, Manchester, M30 0LX, United Kingdom; 2 Faculty of Life Sciences, The University of Manchester, Michael Smith Building, Oxford Road, Manchester, M13 9PT, United Kingdom; Institute of Microbiology, SWITZERLAND

## Abstract

Antifungal drugs acting via new mechanisms of action are urgently needed to combat the increasing numbers of severe fungal infections caused by pathogens such as *Candida albicans*. The phosphopantetheinyl transferase of *Aspergillus fumigatus*, encoded by the essential gene *pptB*, has previously been identified as a potential antifungal target. This study investigated the function of its orthologue in *C*. *albicans*, *PPT2*/C1_09480W by placing one allele under the control of the regulatable *MET3* promoter, and deleting the remaining allele. The phenotypes of this conditional null mutant showed that, as in *A*. *fumigatus*, the gene *PPT2* is essential for growth in *C*. *albicans*, thus fulfilling one aspect of an efficient antifungal target. The catalytic activity of Ppt2 as a phosphopantetheinyl transferase and the acyl carrier protein Acp1 as a substrate were demonstrated in a fluorescence transfer assay, using recombinant Ppt2 and Acp1 produced and purified from *E*.*coli*. A fluorescence polarisation assay amenable to high-throughput screening was also developed. Therefore we have identified Ppt2 as a broad-spectrum novel antifungal target and developed tools to identify inhibitors as potentially new antifungal compounds.

## Introduction

The human burden of fungal disease is immense with approximately 1.7 billion people affected by fungal infections worldwide per annum. Of these, invasive fungal infections are estimated to be the cause of approximately 1.5 million deaths per year making this a significant health problem [1]. *Candida spp*. are opportunistic pathogens and the most common cause of all the human mycoses. They are commensal organisms that form part of the normal human flora and reside in the mucous membranes of the mouth, vagina or digestive tract in a majority of the population. However, an increase in vulnerable patients including the immunocompromised, transplant recipients and intensive care patients has caused a significant increase in the incidences of invasive fungal infections caused by *Candida spp*. Disseminated invasive candidiasis in particular is becoming a serious health problem in the developed world with *Candida albicans* causing around 400,000 life-threatening infections worldwide [1].

There are 3 main classes of antifungal drugs: polyenes, ergosterol biosynthesis inhibitors and echinocandins. Many issues exist with these treatments including the emergence of resistance, drug-drug interactions and toxicity. Additionally, even with the best available treatment, patients with invasive candidiasis have a reported mortality rate of between 30%– 90% [2–4]. Therefore, new classes of antifungals with alternative mechanisms of action are required to address this need.

The products of genes that are essential for growth have been proposed as good antifungal drug targets [5–7]. Inhibitors of such targets should, by definition, render the organism inviable. It should be feasible to identify broad spectrum drug targets where the essential gene is present in multiple fungal species, depending on the protein sequence homology between species, and the nature of the drug-target interaction.

The 4’-phosphopantetheine (4’PPT) portion of coenzyme A (CoA) is an essential group for many carrier proteins and enzymes. Addition of this group is required for the correct function of polyketide synthase (PKS), non-ribosomal peptide synthetase (NRPS) and fatty acid synthase (FAS). The 4’PPT group is transferred to a highly conserved serine motif in the acceptor protein in a magnesium dependent reaction [8] by phosphopantetheinyl transferases (PPTases). The terminal thiol group of the 4’PPT is the site at which elongation occurs via thioester linkages and attachments are covalently linked [9].

Phosphopantetheinyl transferases are found in bacterial, fungal and mammalian cells. In fungal genomes there are three types of PPTase. The first is integrated within the cytoplasmic fatty acid synthase and transfers the 4’PPT group to an acyl carrier protein (ACP) domain within the same protein. The second (Lys5 in *Saccharomyces cerevisiae* and *C*. *albicans*; PptA in *A*. *fumigatus*) is involved in lysine biosynthesis, activating α-aminoadipate reductase [10–13] with further functions including activation of PKS and NRPS. The third PPTase, (Ppt2 in *S*. *cerevisiae*; PptB in *A*. *fumigatus*) is involved in mitochondrial fatty acid synthesis, and is required to transfer the 4’PPT from CoA to the mitochondrial acyl carrier protein Acp1. Previous work has demonstrated that in *A*. *fumigatus* the gene encoding PptB is essential for viability [10].

In contrast, in humans, only one type of PPTase has been identified. It is a broad spectrum PPTase which is able to phosphopantetheinylate the ACP components of both cytosolic and mitochondrial FAS systems, as well as the aminoadipate semialdehyde dehydrogenase, associated with lysine degradation [14]. This human PPTase aligns most closely to the Sfp-type PPTases, whereas fungal mitochondrial PPTases, such as PptB, are more similar to the structurally distinct AcpS-type of PPTase. This suggests the possibility that mitochondrial PPTases would make selective targets for which fungal-specific inhibitors could be identified.

In this study we have evaluated the suitability of a putative *C*. *albicans* PPTase as an antifungal target, investigating its essentiality and developing an assay suitable for high-throughput screening of potential inhibitors for use as antifungals.

## Materials and Methods

### Bioinformatics

BLASTP analysis of the *C*. *albicans* genome was used to identify the orthologue of the phosphopantetheinyl transferase Ppt2/PptB using *S*. *cerevisiae* Ppt2 and *A*. *fumigatus* PptB as probes, and also the homologues of *S*. *cerevisae* Acp1 and *A*. *fumigatus* AcpA. Sequences were acquired from the Candida Genome Database (http://www.candidagenome.org/) or NCBI reference sequence database (http://www.ncbi.nlm.nih.gov/) for all species. The sequences in fasta format were aligned in ClustalW (http://embnet.vital-it.ch/software/ClustalW.html) and the ALN format output was annotated using Boxshade (http://embnet.vital-it.ch/software/BOX_form.html).

### Strains and growth media

A list of *C*. *albicans* strains used and generated in this study is given in Table 1. *C*. *albicans* strains were grown in synthetic dextrose media (1 X Yeast Nitrogen Base with 5% ammonium sulphate (Formedium); 2% glucose) supplemented with 20 mg/L L-arginine, 20 mg/L L-histidine and 20 mg/L uridine, as appropriate. Where downregulation of the *MET3* promoter was required, methionine and cysteine were added at a final concentration of 2.5 mM each [15]. *C*. *albicans* strains were grown at 30°C unless otherwise stated.

**Table 1:** *C*. *albicans* strains used in this study.

Strain	Genotype	Description	Reference
SN76	*arg4*Δ/*arg4*Δ *his1*Δ/*his1*Δ *ura3*Δ::*imm434/ura3*Δ::*imm434 iro1*Δ::*imm434*/*iro1*Δ:: *imm434*	Parental auxotrophic strain	[16]
KDP1	*arg4*Δ/*arg4*Δ *his1*Δ/*his1*Δ *ura3*Δ::*imm434/ura3*Δ::*imm434 iro1*Δ::*imm434*/*iro1*Δ:: *imm434 PPT2/pMET3-PPT2*::*URA3*	Conditional heterozygote: one wild type *PPT2* allele; one *PPT2* allele under control of *MET3* promoter	This study
KDP2 and KDP3	*arg4*Δ/*arg4*Δ *his1*Δ/*his1*Δ *ura3*Δ::*imm434/ura3*Δ::*imm434 iro1*Δ::*imm434*/*iro1*Δ:: *imm434 ppt2*Δ::*ARG4/pMET3-PPT2*::*URA3*	Conditional PPT2 null mutant: one *PPT2* allele knocked out; one *PPT2* allele under control of *MET3* promoter	This study

### DNA manipulations

*C*. *albicans* genomic DNA was isolated and RNase-treated using the MasterPure Yeast DNA Purification kit (EPICENTRE Biotechnologies) following the manufacturer’s instructions. Typically PCR reactions were carried out in a volume of 50 μl with reaction mixes containing 1 unit/μl KOD Hot Start DNA polymerase (Novagen), 0.2 mM dNTPs, 1.5 mM MgSO_4_, 10 pmol primers, 100 ng genomic DNA or 20 ng plasmid DNA in 1x KOD DNA polymerase buffer. Transformation of *C*. *albicans* was performed using the method described by Walther and Wendland [17]. Typically 1–5 μg transforming DNA was used for each reaction.

### Generation of DNA manipulation constructs

#### *PPT2* promoter replacement construct

The promoter replacement constructs were designed to insert a *URA3* marker followed by the *MET3* promoter upstream of the gene of interest. These were made by fusion PCR (FPCR) [18]. Firstly, three PCR products were prepared corresponding to the genomic target insertion sites and the URA3MET3 promoter replacement section. A 391 bp region upstream of the start codon of *PPT2* was amplified from *C*. *albicans* SN76 genomic DNA using primers KDPF and KDPMR (KDPMR included 25 bp of the URA3MET3 region as an overlap for the FPCR; see S1 Table for primer sequences). The same DNA template was used to amplify a 394 bp region downstream from the start codon of PPT2 with primers KDPMF and KDPR (KDPMF included 25bp of the URA3MET3 region as an overlap for the FPCR). Lastly, the URA3MET3 region was amplified from the pMET3 plasmid using primers KDMF and KDMR. The PCR products were purified and subsequently annealed in a final fusion PCR reaction using primers KDPF and KDPR to amplify the entire promoter replacement construct (3518 bp). The identity of the PCR product was confirmed by restriction endonuclease digests using *Eco*R1 and *Vsp*1 following gel-purification of the FPCR product.

#### *PPT2* deletion construct

Long primers KDPAF and KDPAR, each with 100 bp homology to target insertion sequences, were used to amplify the *ARG4* marker using the pLAL plasmid as template DNA. This plasmid is based on the shuttle vector pRS513 and contains the cassette loxP-CaARG4-loxP [19, 20]. The insert was designed to delete the majority of the *PPT2* ORF from 42 bp downstream from the ATG start codon until 408 bp, at the end of the gene, and also allowed for transformants to be isolated on selective media (minus arginine).

### Construction of conditional null *PPT2* mutants of *C*. *albicans*

**Fig 1:**
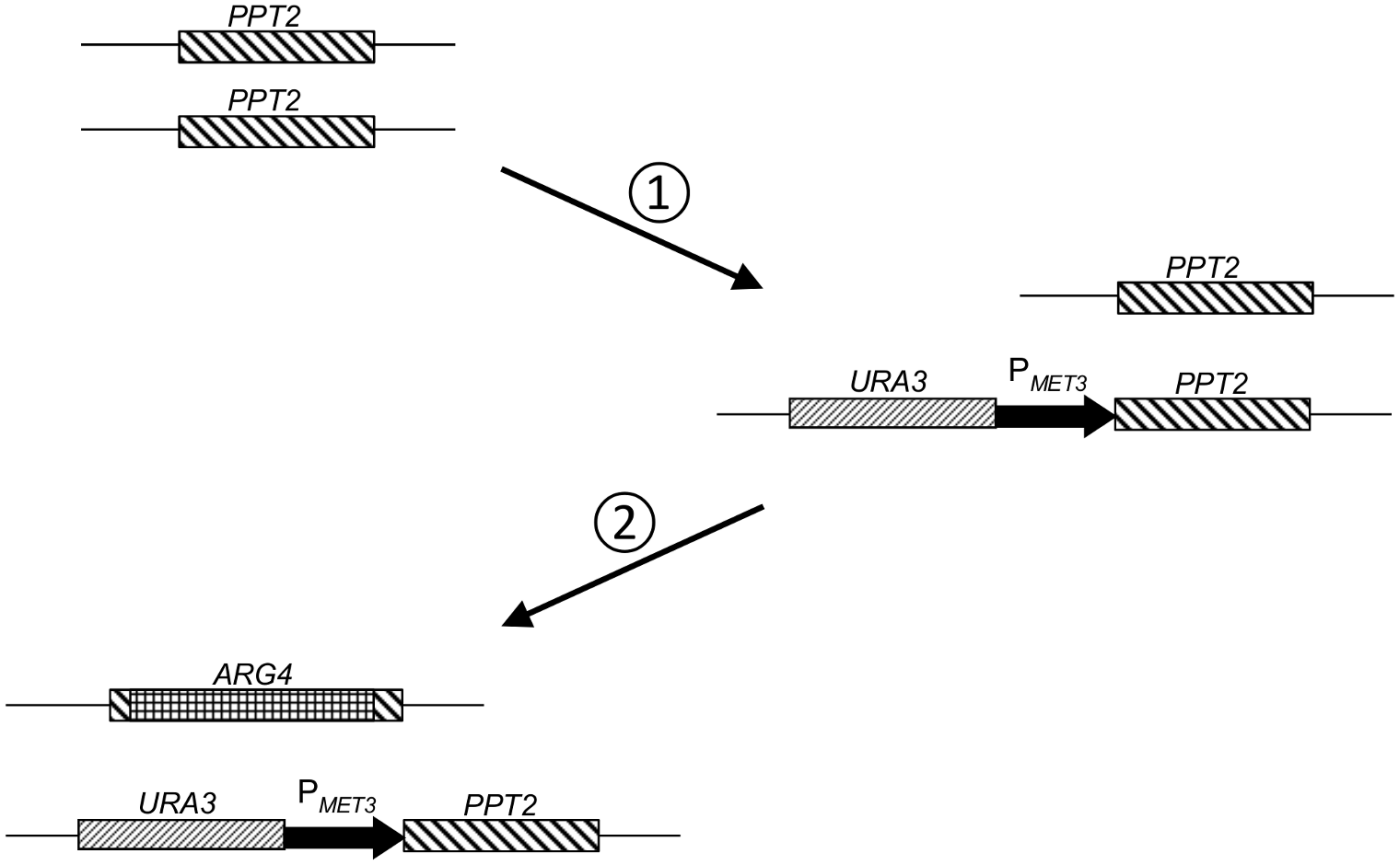
Generation of conditional null *PPT2* mutants of *C*. *albicans*. Step 1: Promoter replacement—*MET3* promoter introduced 5’ of the start of the *PPT2* coding region of one allele with *URA3* as a selection marker. Step 2: Remaining wild type *PPT2* gene knocked out by replacement of the majority of the coding sequence with the *ARG4* gene.

Two steps were involved in constructing conditional *PPT2* mutants using *C*. *albicans* SN76 as the parental strain. In the first, one allele of *PPT2* was placed under the control of the *MET3* promoter using the FPCR product (described above) containing the *URA3* marker (Fig 1, step 1). In the second step the majority of the ORF of the remaining allele of *PPT2* was deleted using the deletion construct described above (Fig 1, step 2).

*C*. *albicans* SN76 was transformed using the lithium acetate method described by Walther and Wendland [17]. Putative transformants were selected on appropriate SD selective media: without uridine in the first step and without arginine, in the second step, respectively. Diagnostic PCR was performed on ten colonies to confirm the site of promoter replacement using primers KDMDF and KDPDR to identify the correct promoter insertion (PCR product size 1310 bp). The majority of colonies tested in the colony PCR proved positive for the promoter replacement insertion. One of the transformants that tested positive for the promoter replacement insertion, named KDP1 was taken further in a second round of transformation using the gene deletion construct. Transformants isolated on selective media were analysed by diagnostic PCR (S1A–S1C Fig; primers indicated on figure), and two strains, KDP2 and KDP3, confirmed as conditional *PPT2* null mutants were taken for further tests.

#### Analysing conditional null mutant’s phenotypes

A single colony of the desired *C*. *albicans* strain was used to inoculate 3 ml aliquots of SD media and incubated overnight at 30°C. The OD_600_ of an overnight culture of each strain was measured and adjusted to 0.1 in fresh SD media. A fivefold serial dilution was performed in dH_2_O. Aliquots of 5μl of each sample were spotted onto SD agar with and without the presence of 2.5 mM methionine and 2.5 mM cysteine and incubated at 30°C for 4 days.

### Protein expression and purification

No introns or CTG codons were predicted for the *PPT2*, *ACP1* and *ACP12* sequences, so genomic DNA was used as the template for amplification of DNA for protein expression. The following primer pairs were used for PCR amplification: CAPPT2F and CAPPT2R for *PPT2*; CAACP1F and CAACP1R for *ACP1*; CAACP12F and CAACP12R for *ACP12* (S1 Table for primer sequences). Truncated versions of the acyl carrier proteins lacking the N-terminal mitochondrial sequences (predicted by the program TargetP to be cleaved in the mature protein) were expressed. The predicted cleavage sites align with the predicted cleavage site for *A*. *fumigatus* AcpA. The purified PCR products were cloned into pET43.1 (*PPT2*) or pET30 (*ACP1* and *ACP12*). The proteins were expressed in *E*. *coli* BL21 (DE3) upon induction with 0.5 mM IPTG. The proteins were purified by immobilised metal affinity chromatography (IMAC) using HisBind resin (Novagen) following the manufacturer’s instructions.

### Fluorescent labelling and transfer

In order to monitor the transfer of a phosphopantetheine group from coenzyme A to a target protein, a fluorescent dye BODIPY TMR maleimide (Life Technologies) was attached to the terminal sulphydryl group of coenzyme A [21]. CoA was labelled with BODIPY TMR maleimide as described previously [10]. Essentially, 1.5 mM BODIPY TMR was incubated with 1 mM CoA in 50% DMSO, 50 mM Tris-HCl, 5 mM MgCl_2_, pH 7.5 on ice for 30 minutes, then at room temperature for 10 minutes. Excess unbound BODIPY TMR was extracted with ethyl acetate. This extraction was repeated until the ethyl acetate remained clear and did not fluoresce under UV light. In a typical reaction, the recombinant PPTase enzyme, Ppt2p (500 ng), was mixed with 5 μg Acp1p or 5 μg Acp12p and 0.1 volumes of a 1:10 dilution of the Bodipy TMR-labelled CoA (CoA-BTMR) in an assay buffer of 62.5 mM Tris, 12.5 mM MgCl_2_ pH 6.75. The reaction proceeded at room temperature for 1 hour before separation by polyacrylamide gel electrophoresis.

### Fluorescence polarisation (FP) based assay

Typical reaction components were 2–8 ng/μl PPTase, 30 ng/μl ACP and 1:25 CoA-BTMR in the assay buffer described above. Where EDTA was added, this was at a final concentration of 20 mM. Reagents were mixed in 384-well black plates and left at room temperature for 0–85 minutes. FP was measured on a PerkinElmer Fusion—Alpha FP HT plate reader using the 540/20FP excitation filter and the 580/15FP emission filter.

To establish the quality of the Ppt2 FP assay two 384-well black microplates were set up with 30 ng/μl Acp1p and 1:25 CoA-BTMR in assay buffer. 1% DMSO was also included as compounds to be tested for inhibition would be solubilised in DMSO. 4 ng/μl Ppt2p was added to the positive control plate and an equivalent volume of buffer added to the negative control plate. The reaction was stopped by addition of EDTA to 20 mM after 40 min and the FP values of the plates were determined. Z’, a measure of the quality of the assay [22], is calculated from the following equation:

$$Z^{\prime }=\;1-\frac{3\sigma_{p}+3\sigma_{n}}{\mu_{p}-\;\mu_{n}}$$

*σ*_*p*_—standard deviation of positive controls; *σ*_*n*_—standard deviation of negative controls; *μ*_*p*_—mean of positive controls; *μ*_*n*_—mean of negative controls.

## Results

### *In silico* identification of *PPT2* and its potential substrates *ACP1* and *ACP12* in *C*. *albicans*

**Fig 2:**
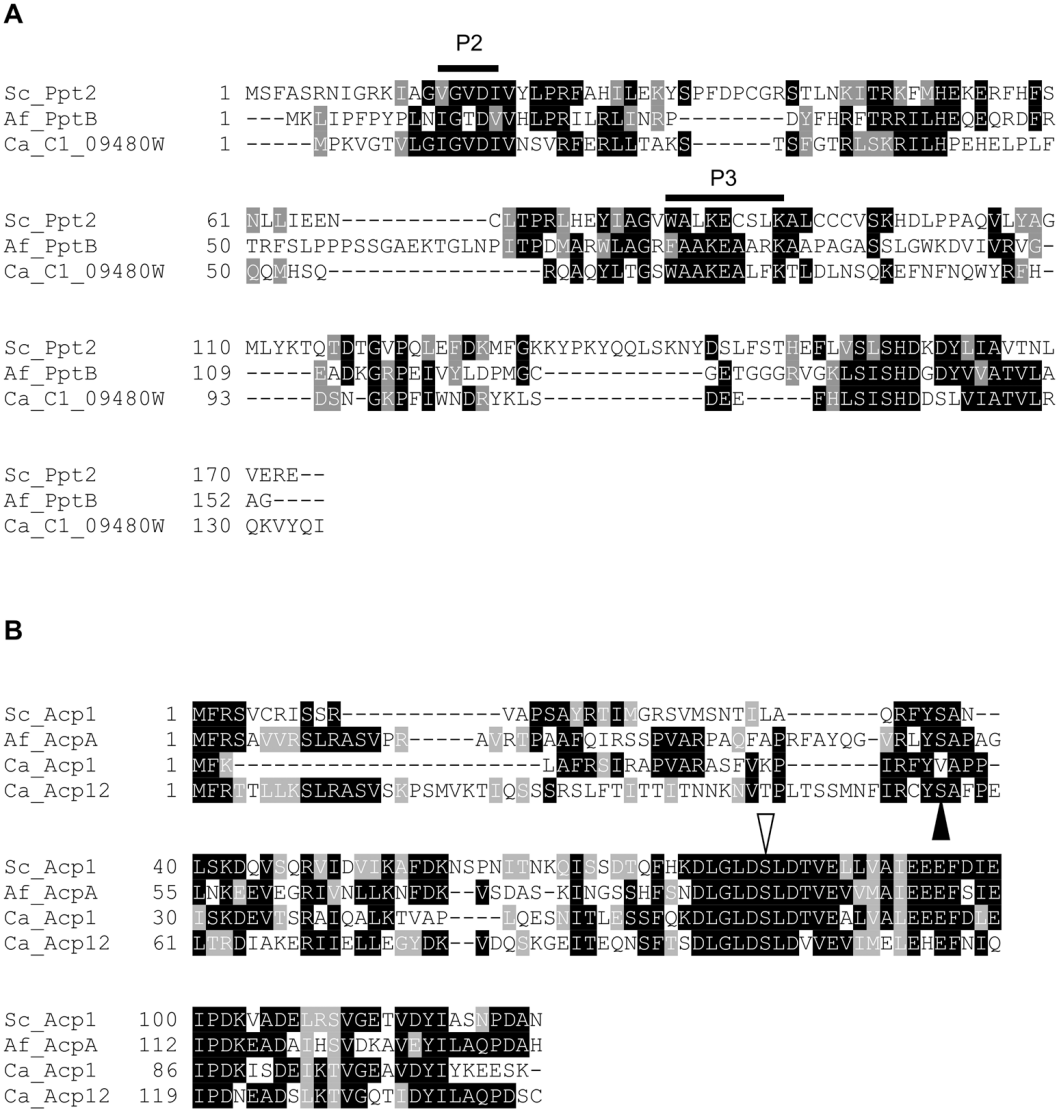
Alignment of fungal phosphopantetheinyl transferases (A) and acyl carrier proteins (B). (A) *S*. *cerevisiae* Ppt2p (Sc_Ppt2), *A*. *fumigatus* PptB (Af_PptB), and *C*. *albicans* C1_09480W/Ppt2p (Ca_C1_09480W). The P2 and P3 regions found in PPTase family enzymes are indicated. (B) *S*. *cerevisiae* Acp1p (Sc_Acp1), *A*. *fumigatus* (Af_AcpA), *C*. *albicans* Acp1p and Acp12p (Ca_Acp1 and Ca_Acp12). The conserved pantetheinylated serine residues are indicated by the open triangle. The recombinant versions of the *A*. *fumigatus* and *C*. *albicans* acyl carrier proteins started at the residue indicated by the closed triangle.

BLASTP analysis of the *C*. *albicans* genome using *S*. *cerevisiae* Ppt2p and *A*. *fumigatus* PptB as probes identified C1_09480W (orf 19.4812) as the nearest homologue (see S2 Table for BLAST analysis results). This gene is predicted to encode a protein of 135 amino acids which has low overall identity to ScPpt2p and PptB (Fig 2A; S2 Table). However two of the most conserved regions between these three sequences correspond to the P2 and P3 regions known to be conserved among the PPTase superfamily (Fig 2A) [8, 9]. Previously, the mitochondrial acyl carrier proteins ScAcp1 and AcpA have been identified as the targets of ScPpt2p and PptB, respectively. BLAST analysis using ScAcp1 and AcpA identified two putative substrates for C1_09480W/*PPT2* in the *C*. *albicans* genome: Acp1 (C1_06060C) and Acp12 (C2_04030C) with a predicted size of 110 and 144 amino acids, respectively (Fig 2B).

### *PPT2* is essential for growth in *C*. *albicans*

Previous work has shown that *pptB* is essential for growth in the human opportunistic pathogen *A*. *fumigatus* [10] making it a potentially good target for novel antifungals. To assess whether this could lead to broader spectrum antifungals it was necessary to confirm whether its orthologue in *C*. *albicans* was also essential for growth. For this purpose, conditional null mutants were constructed by placing one allele of *C*. *albicans PPT2* under the *MET3* regulatable promoter and deleting the remaining allele (Fig 1) as described in Materials and Methods.

**Fig 3:**
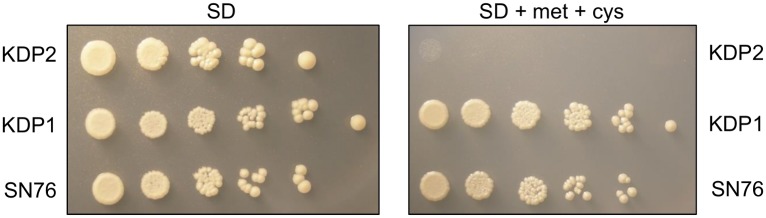
*PPT2* is essential for growth in *C*. *albicans*. Growth phenotypes of *PPT2* conditional null mutant (KDP2), conditional heterozygote (KDP1) and SN76 parental strain in presence and absence of 2.5 mM methionine and 2.5 mM cysteine after 4 days incubation at 30°C.

The mutant strains were grown in the presence or absence of 2.5 mM cysteine and 2.5 mM methionine to down regulate the *MET3* promoter as described by Care et al. [15]. As shown in Fig 3, the conditional heterozygous mutant (KDP1) and the parental strain SN76 were able to grow on SD medium (supplemented with histidine) in repressive conditions. In contrast, the conditional null mutant KDP2 was unable to grow under the same conditions suggesting that *PPT2* is essential for growth in *C*. *albicans*. This was investigated further by replacing the glucose with a variety of carbon sources. The conditional null strains KDP2 and KDP3 were unable to grow on galactose, glycerol, ethanol, lactate, citrate or acetate when *PPT2* was down-regulated (S2 Fig).

### Characterisation of Ppt2p activity using a fluorescence shift assay

**Fig 4:**
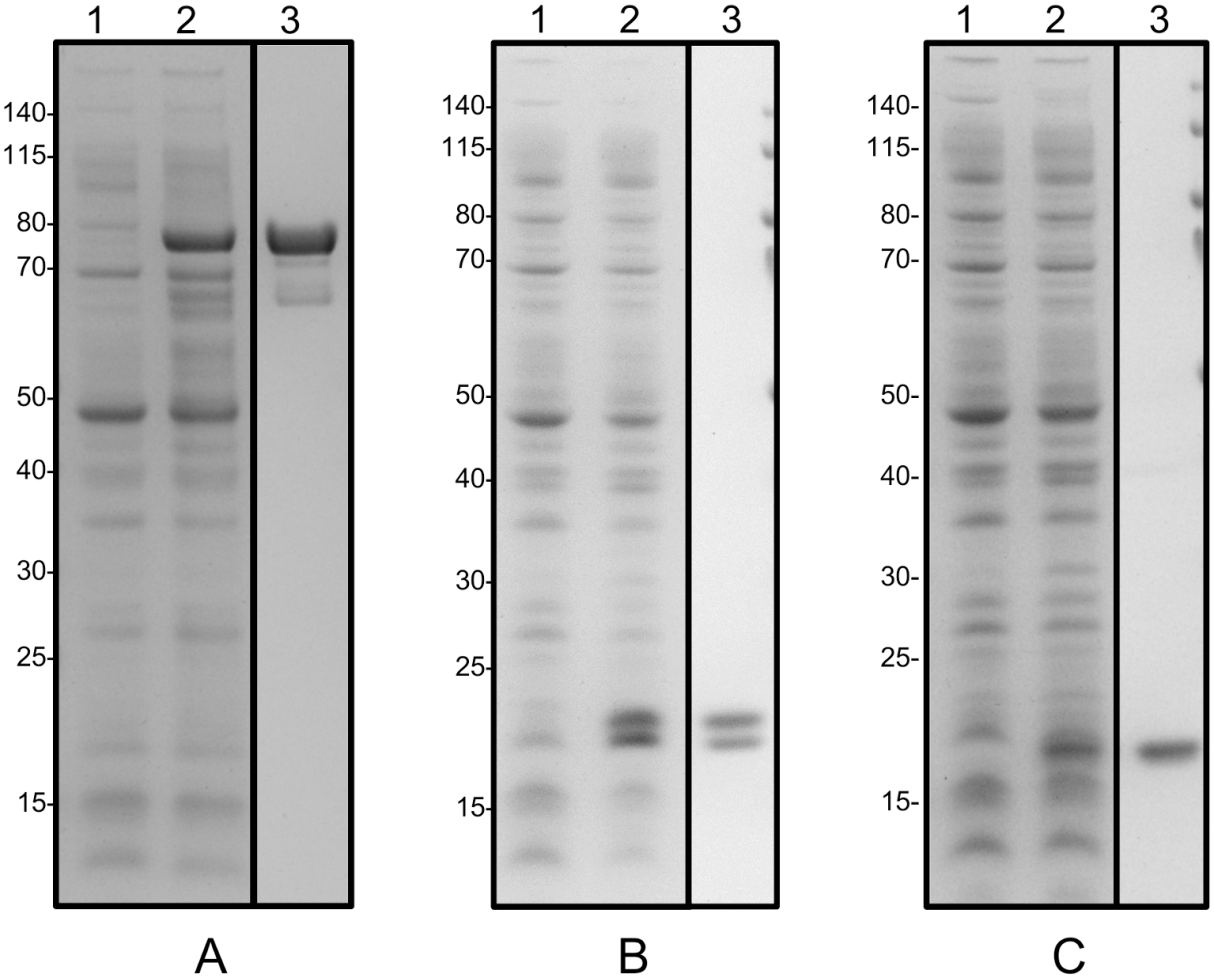
Expression and purification of recombinant *C*. *albicans* Ppt2 and putative acyl carrier protein substrates. *E*. *coli* BL21 DE3 was transformed with (A) pET43.1-Ppt2 (76 kDa), (B) pET30-Acp1 (16 kDa) and (C) pET30-Acp12 (16 kDa) and expression of the recombinant proteins induced with 0.5 mM IPTG. Recombinant proteins were purified by immobilised metal affinity chromatography. Fractions were separated by SDS PAGE. The migration of molecular weight markers is indicated by the figures (kDa) to the left of each panel. Lane 1, *E*. *coli* lysate before induction; Lane 2, *E*. *coli* lysate after induction with 0.5 mM IPTG and 18h incubation; Lane 3, purified and desalted fraction of recombinant protein.

In order to determine the enzymatic activity of *C*. *albicans* Ppt2p, recombinant Ppt2p, Acp1p and Acp12p were produced in *E*.*coli* and purified by IMAC as described in Materials and Methods. The results of these proteins’ production and purification are shown in Fig 4. The native Ppt2p protein was predicted to be 15.8 kDa, however with an additional fusion tag to aid solubility, the predicted molecular weight was 76 kDa. The recombinant purified Acp1p appeared as two bands as shown in Fig 4B, lane 3. A similar doublet has been observed for *A*. *fumigatus* AcpA recombinant protein expressed in *E*.*coli* [10] and the human mitochondrial ACP after heterologous expression in Sf9 cells [23].

**Fig 5:**
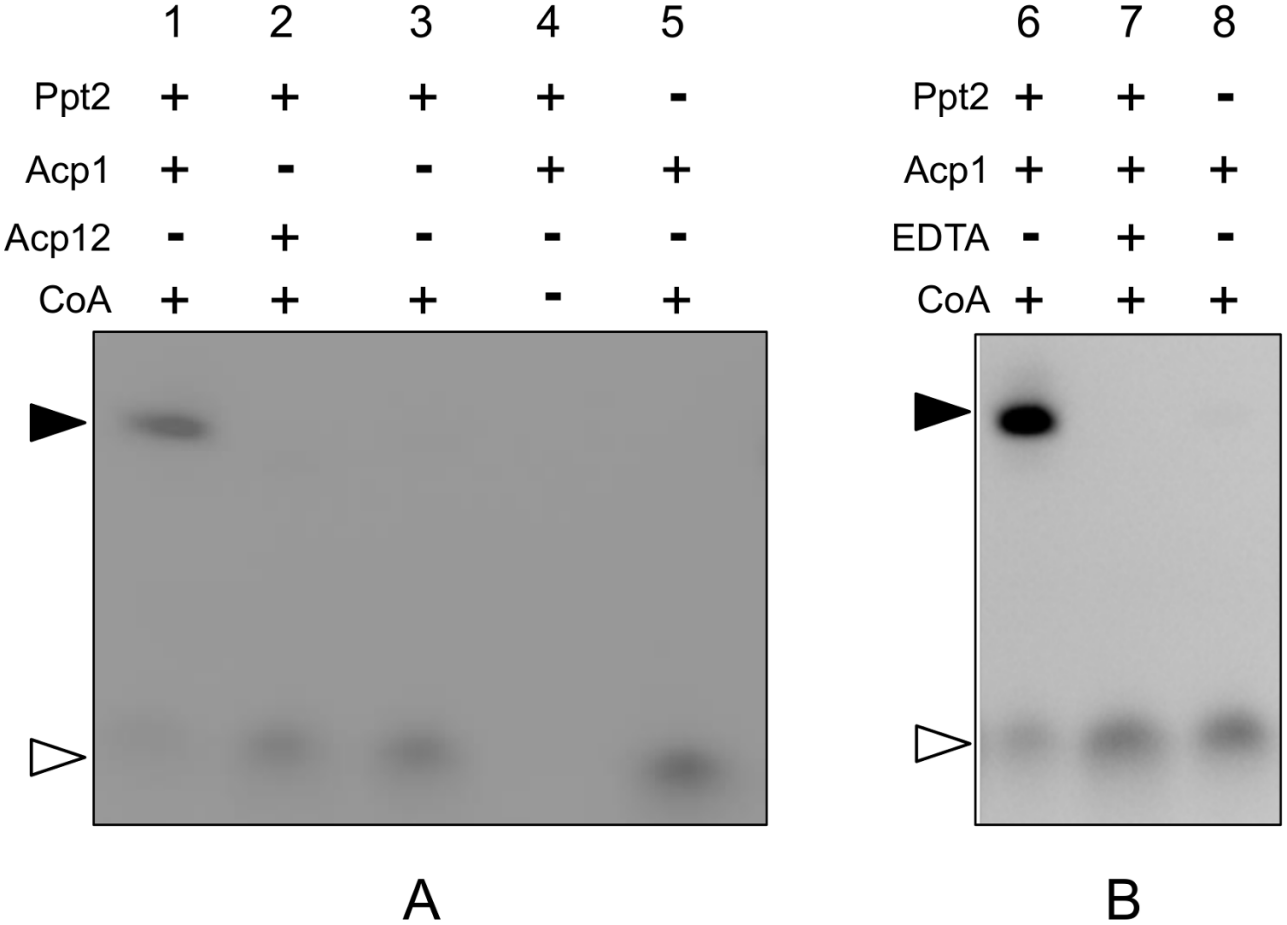
Transfer of fluorescent phosphopantetheine from coenzyme A to ACP. Bodipy TMR-labelled CoA was incubated for 1 hour at room temperature in the presence or absence of 500 ng Ppt2p, 5 μg Acp1p, 5 μg Acp12p and 20 mM EDTA as indicated. The reaction products were then separated by gel electrophoresis and photographed under UV light. (A) Ppt2 transfers a fluorescent phosphopantetheine group from coenzyme A (open arrowhead) to Acp1p (lane 1, closed arrowhead) but not Acp12p (lane 2). (B) Phosphopantetheinylation by Ppt2p requires magnesium—compare the fluorescent product indicated in lane 6 in the presence of 5 mM MgCl_2_ with lane 7 in the presence of 20 mM EDTA.

*C*. *albicans* Ppt2p was predicted to catalyse the transfer of phosphopantetheine from CoA to an ACP substrate in a similar manner to *A*. *fumigatus* PptB and *S*. *cerevisiae* Ppt2p. A fluorescence transfer assay comparable to that described by Allen et al. [10] was used to investigate this PPTase activity. The pantetheine group of coenzyme A was labelled with a fluorescent dye allowing any transfer of the phosphopantetheine group from CoA to ACP to be monitored under UV light. Reaction components were incubated for an hour and separated by gel electrophoresis. Under the conditions of this assay, recombinant Ppt2p was able to catalyse the transfer of the phosphopantetheine group from CoA-BTMR to recombinant Acp1p as a substrate as shown by the fluorescence shift in lanes 1 and 6, Fig 5. However, no fluorescence shift, and hence no phosphopantetheine transfer could be observed when recombinant Acp12p was used as the substrate (lane 2, Fig 5). In the absence of either recombinant Ppt2p (lanes 5 and 8), or Acp1p (lane 3) from the reaction; and alternatively upon addition of EDTA to the reaction (lane 7), no fluorescence shift occurred. These results indicated that Ppt2 catalyses the phosphopantetheine transfer to its substrate Acp1 in a Mg^2+^-dependent manner as is the case for other PPTases.

### Fluorescence Polarisation Assay

The fluorescence transfer assay was successfully used to demonstrate the enzymatic activity of recombinant Ppt2p. However it is not applicable for a high-throughput screening purpose. To address this, an assay based on fluorescence polarisation (FP) was investigated. FP is based on the principle that large fluorescent molecules rotate more slowly than smaller fluorescent molecules, and upon excitation with plane polarised light, emit more light in a fixed plane. The transfer of a fluorophore from a small molecule such as coenzyme A, to a macromolecule, ACP, as in our case, can thus be detected by an increased FP value. An FP-based assay has been successfully used to measure PPTase activity [24–26], and to screen a library of small molecules for inhibitors of the Sfp type bacterial PPTase [27].

**Fig 6:**
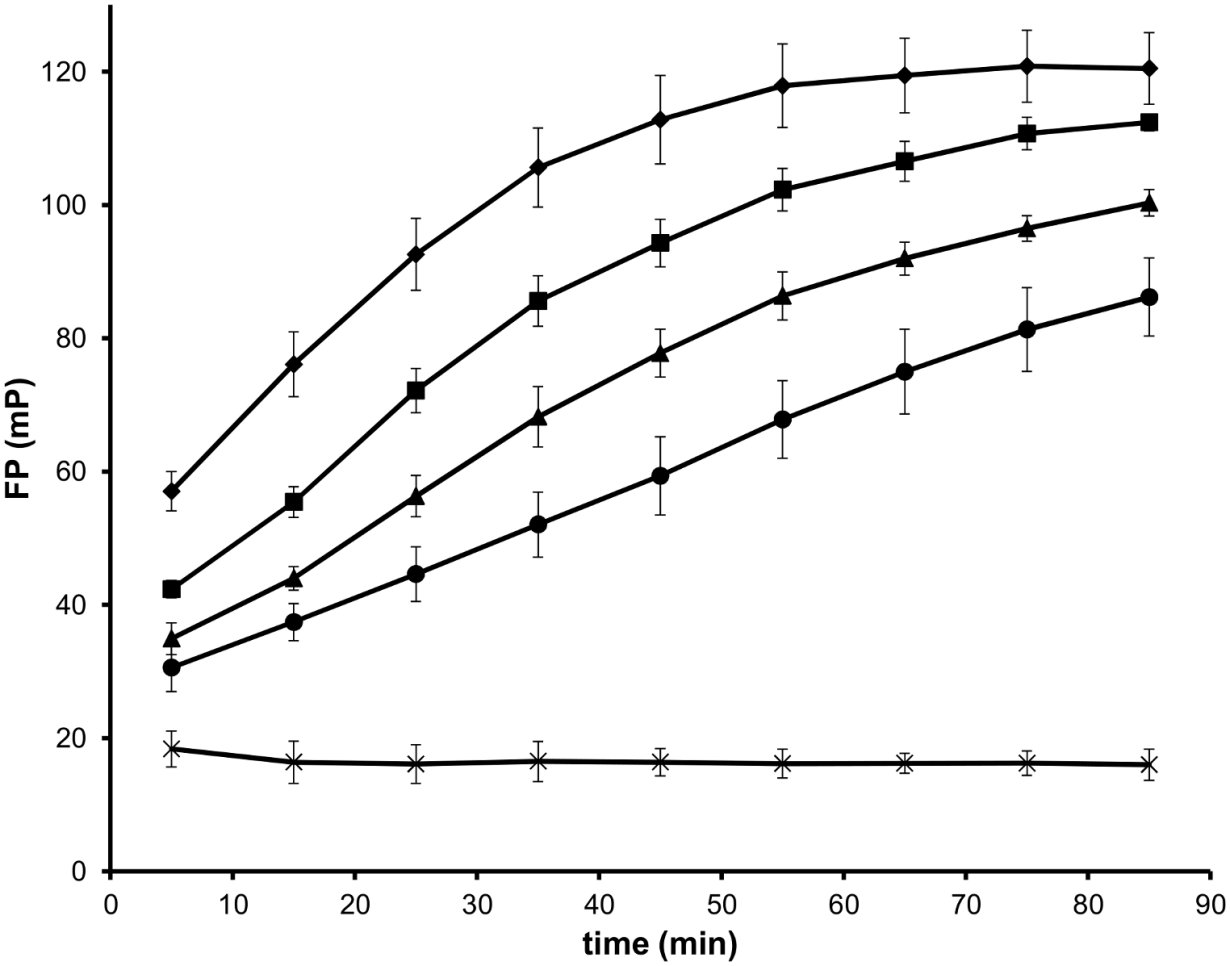
Fluorescence polarisation assay of Ppt2 activity. Recombinant Ppt2p (0 ng/μl, cross; 2 ng/μl, circle; 4 ng/μl, triangle; 6 ng/ul, square; 8 ng/μl, diamond) was combined with 30 ng/μl recombinant Acp1p and CoA-BTMR. FP values were obtained at various time intervals with 3 biological replicates for each data point and the standard deviations displayed on the graph.

In this study FP was applied to follow the PPTase reaction catalysed by recombinant Ppt2p. CoA was labelled with BODIPY—TMR and the transfer of the fluorophore to recombinant Acp1p was monitored as an increase in FP value. In the presence of Ppt2p, its target Acp1p, labelled CoA and magnesium, an increase in FP value was observed compared to a reaction in the absence of Ppt2p (Fig 6). FP values increased with time until the reaction slowed at later timepoints for higher enzyme concentrations. For Ppt2p concentrations up to 8 ng/ μl FP values increased with enzyme concentration during the first hour of incubation. Addition of 20 mM EDTA stopped the reaction progress, allowing multiple plates to be processed, the reaction stopped and read subsequently. As demonstrated for the gel-based fluorescent assay above (Fig 5), EDTA, when added at the start of the reaction, prevented transfer in the FP assay and an IC_50_-like curve was obtained indicating the utility of the screen for identifying PPTase inhibitors (S4 Fig). The quality of the screen was evaluated using positive and negative control plates in the presence and absence of Ppt2p, respectively. Applying the method of Zhang and colleagues [22] a Z’ value of 0.86 was obtained indicating that it is a robust screen. Thus, a suitable high-throughput screen for the identification of inhibitors of *C*. *albicans* Ppt2 has been developed.

## Discussion

Previously, the *A fumigatus* PPTase, PptB was proposed as a promising target for antifungal drug development [10]. In this paper, we have identified C1_09480W as its closest orthologue in *C*. *albicans* and followed convention by appending the name *PPT2* to this gene. By deleting one *PPT2* allele and placing the other under the control of the regulatable promoter *MET3* (Fig 1) we have demonstrated that this gene is essential for growth in *C*. *albicans* (Fig 3).

If the genes encoding homologous proteins are demonstrated to be essential for growth in more than one pathogenic species then these gene products have the potential to be broad-spectrum antifungal targets. For this to result in a broad spectrum drug the inhibitor binding site for multiple species must be similar enough for the inhibitor of one protein to be also effective against its orthologues. Although the overall sequence identity between *C*. *albicans* Ppt2 and *A*. *fumigatus* PptB is low, they contain highly conserved areas (Fig 2), and catalyse the same reaction with target proteins that are also highly similar (Acp1 and AcpA) (Fig 5 and S3 Fig). It may be possible to identify molecules that inhibit both proteins, but that remains to be established. Furthermore, the only human PPTase is of a different structural class [23] improving the chance of finding fungal-specific inhibitors.

This leads us to speculate why Ppt2p is an essential enzyme for growth and hence a good potential antifungal target. Firstly, it was predicted to catalyse the transfer of a phosphopantetheine group from CoA to ACP changing it from an inactive apo to an active holo form. This was shown in the current study by two *in vitro* assays, the gel-based fluoroscence transfer experiment (Fig 5) and by a homogeneous FP assay (Fig 6). In *S*. *cerevisiae* the active mitochondrial ACP, Acp1p, has been shown to be required for lipoic acid synthesis. In yeast, lipoic acid is an important mitochondrial cofactor, essential for mitochondrial respiration. When mitochondrial fatty acid synthesis is disrupted mutants have a respiratory deficient phenotype. Interestingly, *in vitro* studies have shown that addition of lipoic acid to the culture medium of respiratory deficient yeast strains cannot reverse these defects [28, 29]. No other enzyme has been identified in fungi that fulfils the role of phosphopantetheine transfer to the mitochondrial ACP, although in light of the broad specificity of some PPTases, for example the human PPTase [23] this cannot be ruled out. It is also possible that Ppt2p performs other, as yet unknown, functions in the cell that are important for growth.

Unlike *S*. *cerevisiae* and *A*. *fumigatus*, where there appears to be a single mitochondrial acyl carrier protein, two putative mitochondrial ACPs were identified in *C*. *albicans*: Acp1p and Acp12p. Acp1p was identified as a substrate for Ppt2p *in vitro* as demonstrated by the transfer of fluorescently-labelled phosphopantetheine from CoA to Acp1p. Acp12p was not pantetheinylated by Ppt2p under these conditions indicating that it may not be a native substrate for Ppt2p (Fig 5 and S3 Fig). We can rule out misfolding of recombinant Acp12p as a reason for this, because we have observed pantetheinylation of this protein by the *A*. *fumigatus* PptB which appears to be less substrate selective than the *C*. *albicans* orthologue (S3 Fig). Alignment of the two putative *C*. *albicans* ACPs indicates conservation of the phosphopantetheinylated serine within the DSLD motif (Fig 2B). However, Acp12 has a valine, three residues distal to this serine residue, different to the threonine observed in the other ACPs that may be important for activity. Mutation studies around the target serine may shed light on the selectivity observed. Regardless, the fluorescence transfer experiments successfully identified Acp1p as a suitable ACP substrate and confirmed that Ppt2 / C1_09480W was indeed a PPTase.

A fluorescence polarisation assay was developed that proved to be suitable for monitoring the PPTase activity of Ppt2p. This assay is amenable to high-throughput screening to identify inhibitors of Ppt2p. Thus, this study has identified a new antifungal target, *C*. *albicans* Ppt2, and the means to exploit this target to begin a drug discovery programme.

## Supporting Information

S1 Fig Diagnostic PCRs to check correct promoter replacement (A), gene deletion (B) and the presence of wild type allele (C). DNA extracted from the parental strain SN76, the conditional heterozygote KDP1 and the conditional null *PPT2* mutants KDP2 and KDP3, was taken for PCR using the primer pairs indicated in the figure. (A) the correct insertion of the promoter replacement construct was tested at the 5’ end (lanes 1–2) and 3’ end (lanes 4–5). (B) the correct insertion of the *ARG4* cassette for deletion of the remaining *PPT2* allele, was demonstrated by testing the 3’ (lanes 1–2) and 5’ flanking sequences (lanes 4–5) of the disrupted allele. (C) the presence of intact (700 bp) and promoter replaced (3400 bp) *PPT2* genes was tested showing that the intact PPT2 gene was absent in the conditional null strains (KDP2 and KDP3) with the longer product indicating that promoter replacement of *PPT2* had occurred in these strains.
(TIFF)

S2 Fig Growth phenotypes of conditional null mutants on different sources of carbon. Growth phenotypes of *PPT2* conditional null mutants (KDP2 and KDP3), conditional heterozygote (KDP1) and SN76 parental strain in presence and absence of 2.5 mM methionine and 2.5 mM cysteine after 4 days incubation at 30°C. The glucose in the SD medium was replaced by the carbon sources indicated at 2%.
(TIFF)

S3 Fig Selectivity of phosphopantetheinylation of acyl carrier proteins by PptB and Ppt2. PptB from *A*. *fumigatus* (Af-PptB) or *C*. *albicans* Ppt2p (Ppt2) were incubated with CoA-BTMR and *A*. *fumigatus* AcpA (Af-AcpA), *C*. *albicans* Acp1p (Acp1) or *C*. *albicans* Acp12p (Acp12) as indicated for an hour at room temperature. NaCl (500 mM) was included where indicated. Samples were separated by SDS PAGE and the gel exposed to UV. Af-PptB and Af-AcpA were prepared as described in Allen et al. 2011 [10].
(TIFF)

S4 Fig Inhibition of Ppt2 activity by EDTA. 2 ng/μl Ppt2p was incubated in the presence of 30 ng/μl Acp1p and increasing concentrations of EDTA. Transfer of fluorescent phosphopantetheine group from Bodipy-TMR-CoA to Acp1p was determined by fluorescence polarisation at 40 min. Inhibition of transfer was calculated as a percentage of no-EDTA controls and fitted to a sigmoidal curve in XL-fit (IDBS) (n = 4, +/- standard deviation).
(TIF)

S1 Table Primers used in this study. (DOCX)

S2 Table BLASTP analysis of fungal PPTases and their target acyl carrier proteins. The indicated sequences were subjected to BLASTP analysis on the Candida Genome Database. Default settings were used except when indicated by ^&^, where the expect threshold was increased to 100. This result was then confirmed for AfPptB using NCBI BLASTP (default settings) where the same top hit was observed with very similar scores (score 80; Bit score 35.4; E value 2e^-2^). *, Ppt2 name given in this study.
(DOCX)
