# Supplementary figures and images for: Characterisation of the Candida albicans Phosphopantetheinyl Transferase Ppt2 as a Potential Antifungal Drug Target
Source: PLoS One. 2015 Nov 25;10(11):e0143770. doi: 10.1371/journal.pone.0143770 (PMC4659657; doi:10.1371/journal.pone.0143770)

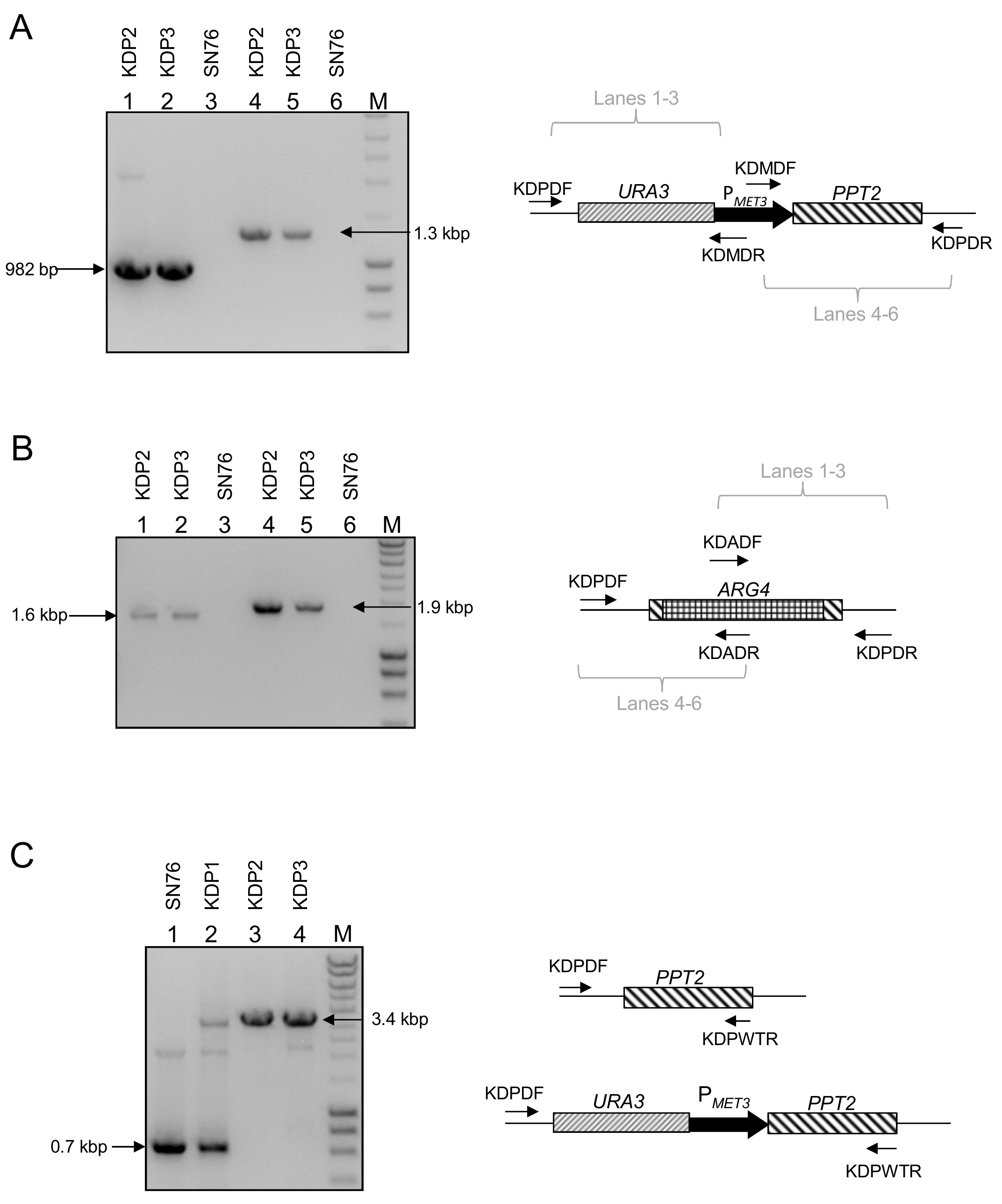

Supplement: S1 Fig — DNA extracted from the parental strain SN76, the conditional heterozygote KDP1 and the conditional null PPT2 mutants KDP2 and KDP3, was taken for PCR using the primer pairs indicated in the figure. (A) the correct insertion of the promoter replacement construct was tested at the 5’ end (lanes 1–2) and 3’ end (lanes 4–5). (B) the correct insertion of the ARG4 cassette for deletion of the remaining PPT2 allele, was demonstrated by testing the 3’ (lanes 1–2) and 5’ flanking sequences (lanes 4–5) of the disrupted allele. (C) the presence of intact (700 bp) and promoter replaced (3400 bp) PPT2 genes was tested showing that the intact PPT2 gene was absent in the conditional null strains (KDP2 and KDP3) with the longer product indicating that promoter replacement of PPT2 had occurred in these strains. (TIFF) [file pone.0143770.s001.tiff]

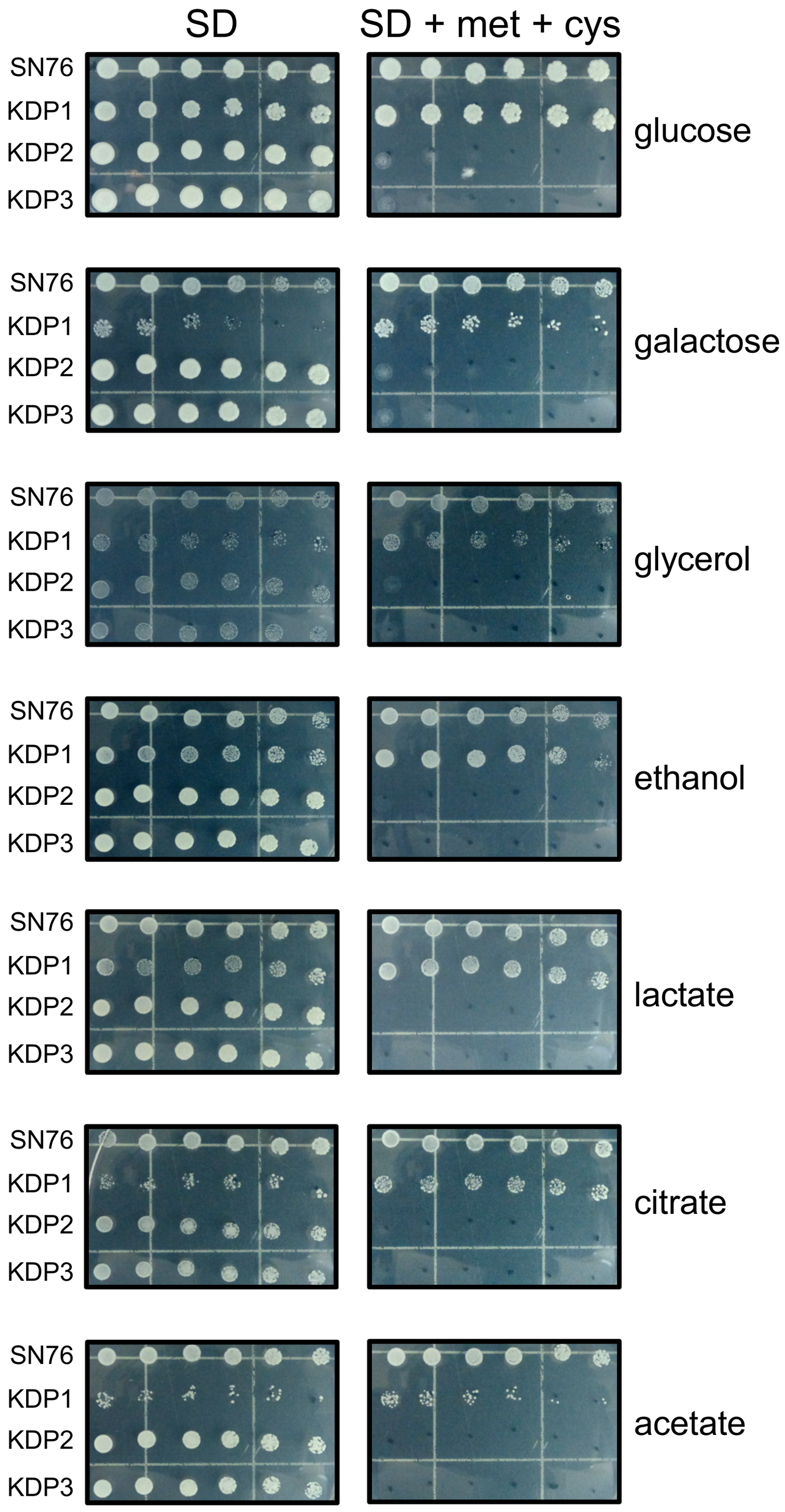

Supplement: S2 Fig — Growth phenotypes of PPT2 conditional null mutants (KDP2 and KDP3), conditional heterozygote (KDP1) and SN76 parental strain in presence and absence of 2.5 mM methionine and 2.5 mM cysteine after 4 days incubation at 30°C. The glucose in the SD medium was replaced by the carbon sources indicated at 2%. (TIFF) [file pone.0143770.s002.tiff]

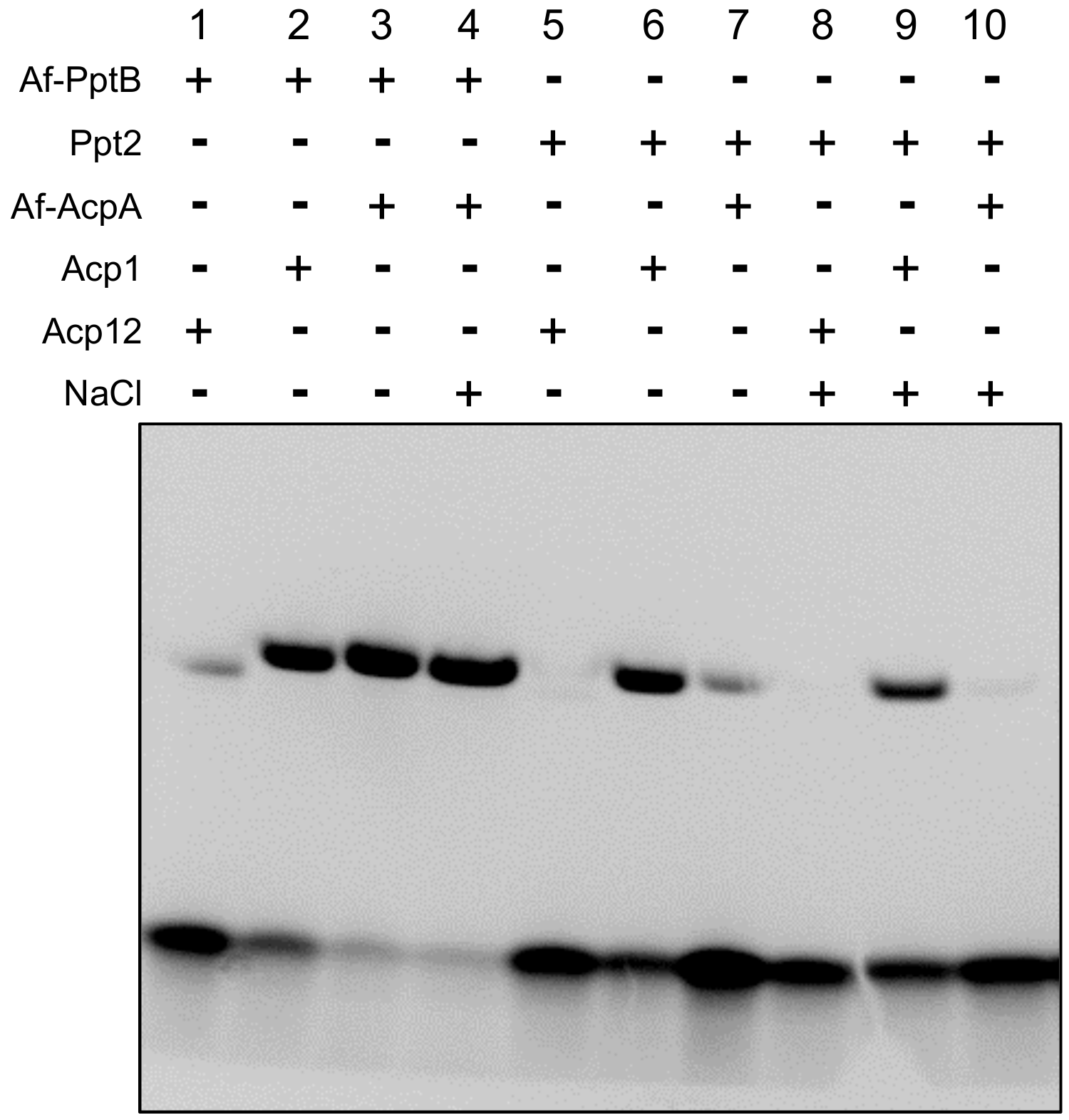

Supplement: S3 Fig — PptB from A. fumigatus (Af-PptB) or C. albicans Ppt2p (Ppt2) were incubated with CoA-BTMR and A. fumigatus AcpA (Af-AcpA), C. albicans Acp1p (Acp1) or C. albicans Acp12p (Acp12) as indicated for an hour at room temperature. NaCl (500 mM) was included where indicated. Samples were separated by SDS PAGE and the gel exposed to UV. Af-PptB and Af-AcpA were prepared as described in Allen et al. 2011 [10]. (TIFF) [file pone.0143770.s003.tiff]

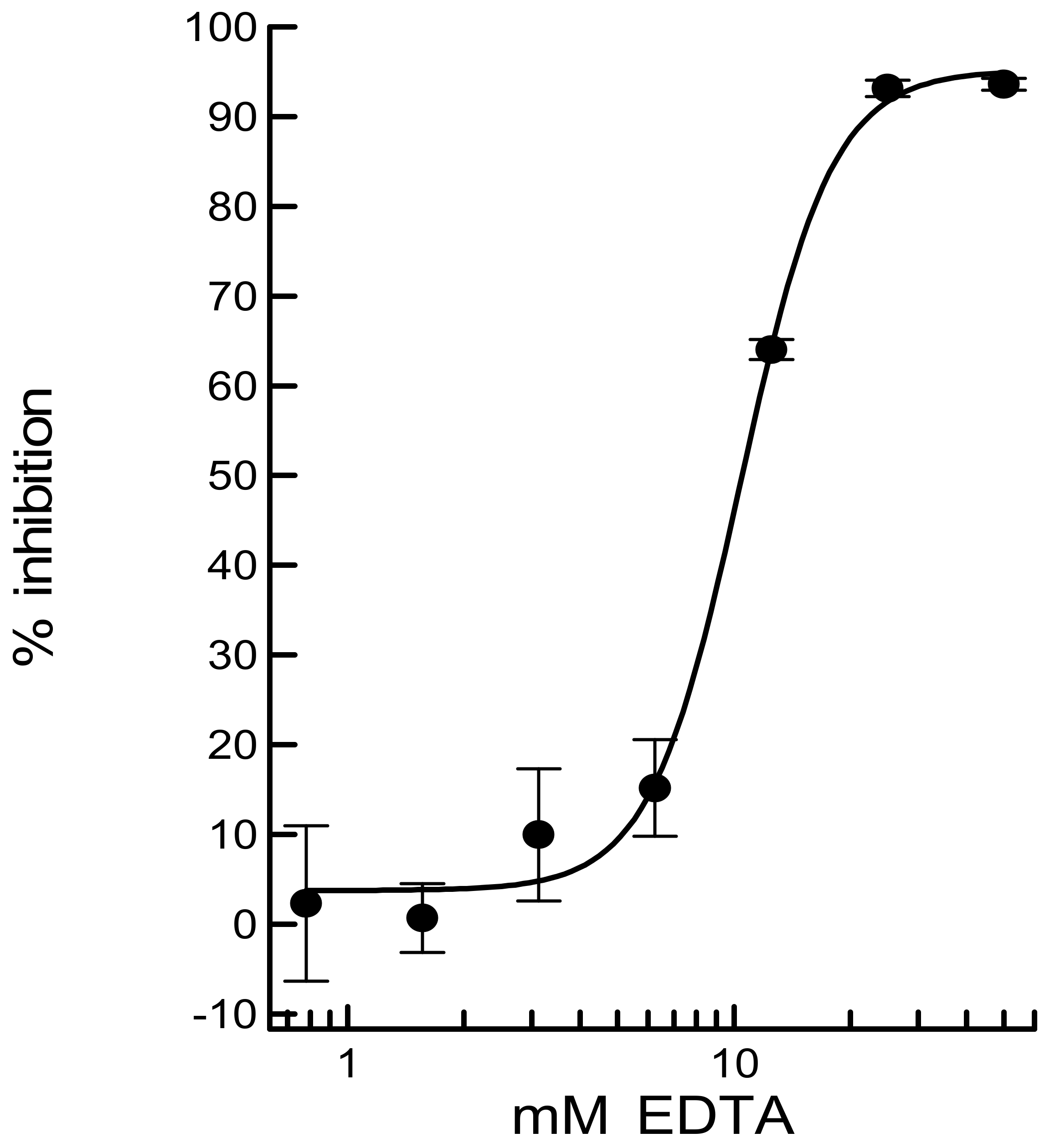

Supplement: S4 Fig — 2 ng/μl Ppt2p was incubated in the presence of 30 ng/μl Acp1p and increasing concentrations of EDTA. Transfer of fluorescent phosphopantetheine group from Bodipy-TMR-CoA to Acp1p was determined by fluorescence polarisation at 40 min. Inhibition of transfer was calculated as a percentage of no-EDTA controls and fitted to a sigmoidal curve in XL-fit (IDBS) (n = 4, +/- standard deviation). (TIF) [file pone.0143770.s004.tif]
