# Supplementary material for: Characterisation of the Candida albicans Phosphopantetheinyl Transferase Ppt2 as a Potential Antifungal Drug Target
Source: PLoS One. 2015 Nov 25;10(11):e0143770. doi: 10.1371/journal.pone.0143770 (PMC4659657; doi:10.1371/journal.pone.0143770)
Supplement: S1 Table — (DOCX) [file pone.0143770.s005.docx]

| **Primer Name** | **Sequence (5’ – 3’)** (All overlapping sequences for FPCR or plasmid binding sites underlined) | **Purpose** |
| --- | --- | --- |
| KDMF | CGGCCGACTTGGCCAAGCCTAGATC | Amplifying URA3MET3 region from pMET3 |
| KDMR | TGGGGAGGGTATTTACTTTTAAATA | Amplifying URA3MET3 region from pMET3 |
| KDPF | CACGGTTTC ACC AGTGTC TG | Amplifying region upstream of *PPT2* gene from *C. albicans* genomic DNA /Amplifying whole *PPT2* promoter replacement fusion construct in a FPCR reaction |
| KDPMR | GATCTAGGCTTGGCCAAGTCGGCCGCTGGAAAAATTTCCCCGAGA | Amplifying region upstream of *PPT2* for FPCR from *C. albicans* genomic DNA |
| KDPMF | TATTTAAAAGTAAATACCCTCCCCAATGCCAAAAGTAGGCACTGT | Amplifying start of *PPT2* gene for FPCR from *C. albicans* genomic DNA |
| KDPR | CCTTCTGACGAAGTACTGTAGCAA | Amplifying start of *PPT2* gene from *C. albicans* genomic DNA /Amplifying whole *PPT2* promoter replacement fusion construct in a FPCR reaction |
| KDPAR | CTTTAGATTTGATAAACCTTCTGACGAAGTACTGTAGCAATTACAAGAGAATCATCATGTGAGATACTAAGATGGAACTCTTCATCAGACAATTTGTATCACTAAAGGGAACAAAAGC | Amplifying *ARG4* with long primers targeting *PPT2* from pLAL plasmid |
| KDPAF | GGAAATTTTTCCAGCATCGAGTTAGTAGCTCTCTGTACCTTAATATCTACTACATGTGATGCCAAAAGTAGGCACTGTATTGGGTATAGGTGTTGATATCCCAGGGTTTTCCCAGTCACG | Amplifying *ARG4* with long primers targeting *PPT2* from pLAL plasmid |
| KDMDF | attgctgtggatcacgtgc | To identify correct insertion of *URA3MET3* construct |
| KDMDR | TGCTACTGGTGAGGCATGAG | To identify correct insertion of *URA3MET3* construct |
| KDADF | gcaattcttgaacgagcaca | To identify correct insertion of *ARG4* construct |
| KDADR | gcccatctaataggttgagc | To identify correct insertion of *ARG4* construct |
| KDPDF | GCTGTTCCCAAGTTTGGTGT | To identify correct insertion of *URA3MET3* and *ARG4* constructs in *PPT2* mutants |
| KDPDR | CAAGACCCATCACAATGTCG | To identify correct insertion of *URA3MET3* and *ARG4* constructs in *PPT2* mutants |
| KDPWTR | TGCATCTGCTGAAACAAAGG | For identifying presence of wild type in *PPT2* mutants |
| CAPPT2F | GACGACGACAAGATGCCAAAAGTAGGCACTGTAT | Amplifying *PPT2* for protein expression |
| CAPPT2R | GAGGAGAAGCCCGGTTTAGATTTGATAAACCTTCT | Amplifying *PPT2* for protein expression |
| CAACP1F | gacgacgacaagatgGTTGCCCCACCAATTTC | Amplifying *ACP1* for protein expression |
| CAACP1R | GAGGAGAAGCCCGGTTTATTTAGATTCTTCTTTGT | Amplifying *ACP1* for protein expression |
| CAACP12F | GACGACGACAAGATGAGTGCCTTCCCAGAATT | Amplifying *ACP12* for protein expression |
| CAACP12R | GAGGAGAAGCCCGGTTTAACAAGAATCTGGTTGAG | Amplifying *ACP12* for protein expression |

**S1 Table. Primers used in this study.**
