# Supplementary material for: Characterisation of the Candida albicans Phosphopantetheinyl Transferase Ppt2 as a Potential Antifungal Drug Target
Source: PLoS One. 2015 Nov 25;10(11):e0143770. doi: 10.1371/journal.pone.0143770 (PMC4659657; doi:10.1371/journal.pone.0143770)
Supplement: S2 Table — The indicated sequences were subjected to BLASTP analysis on the Candida Genome Database. Default settings were used except when indicated by &, where the expect threshold was increased to 100. This result was then confirmed for AfPptB using NCBI BLASTP (default settings) where the same top hit was observed with very similar scores (score 80; Bit score 35.4; E value 2e-2). *, Ppt2 name given in this study. (DOCX) [file pone.0143770.s006.docx]

**S2 Table. BLASTP analysis of fungal PPTases and their target acyl carrier proteins.**
